# Thrust and Hydrodynamic Efficiency of the Bundled Flagella

**DOI:** 10.3390/mi10070449

**Published:** 2019-07-04

**Authors:** Umit Danis, Reza Rasooli, Chia-Yuan Chen, Onur Dur, Metin Sitti, Kerem Pekkan

**Affiliations:** 1 Department of Mechanical Engineering, Carnegie Mellon University, Pittsburgh, PA 15213, USA; 2 Department of Mechanical Engineering, Koc University, Istanbul 34450, Turkey; 3 Department of Mechanical Engineering, National Cheng Kung University, Tainan 701, Taiwan; 4 Department of Biomedical Engineering, Carnegie Mellon University, Pittsburgh, PA 15213, USA; 5 Physical Intelligence Department, Max Planck Institute for Intelligent Systems, Stuttgart 70569, Germany

**Keywords:** flagellar propulsion, bacteria locomotion, microswimmer, particle image velocimetry, computational fluid dynamics

## Abstract

The motility mechanism of prokaryotic organisms has inspired many untethered microswimmers that could potentially perform minimally invasive medical procedures in stagnant fluid regions inside the human body. Some of these microswimmers are inspired by bacteria with single or multiple helical flagella to propel efficiently and fast. For multiple flagella configurations, the direct measurement of thrust and hydrodynamic propulsion efficiency has been challenging due to the ambiguous mechanical coupling between the flow field and mechanical power input. To address this challenge and to compare alternative micropropulsion designs, a methodology based on volumetric velocity field acquisition is developed to acquire the key propulsive performance parameters from scaled-up swimmer prototypes. A digital particle image velocimetry (PIV) analysis protocol was implemented and experiments were conducted with the aid of computational fluid dynamics (CFD). First, this methodology was validated using a rotating single-flagellum similitude model. In addition to the standard PIV error assessment, validation studies included 2D vs. 3D PIV, axial vs. lateral PIV and simultaneously acquired direct thrust force measurement comparisons. Compatible with typical micropropulsion flow regimes, experiments were conducted both for very low and higher Reynolds (Re) number regimes (up to a Re number = 0.01) than that are reported in the literature. Finally, multiple flagella bundling configurations at 0°, 90° and 180° helical phase-shift angles were studied using scaled-up multiple concentric flagella thrust elements. Thrust generation was found to be maximal for the in-phase (0°) bundling configuration but with ~50% lower hydrodynamic efficiency than the single flagellum. The proposed measurement protocol and static thrust test-bench can be used for bio-inspired microscale propulsion methods, where direct thrust and efficiency measurement are required.

## 1. Introduction

The vast biodiversity of swimming microorganisms [1,2], whose survival depends on fluidic propulsion, has inspired a wide range of synthetic microswimmers in the recent decade towards biomedical and environmental applications [3,4,5,6,7,8,9,10,11,12,13,14,15,16]. Particularly prokaryotic microorganisms and bacteria, such as *Escherichia coli*, which have bidirectional molecular motors embedded in a cell membrane to generate rotational torque on their multiple flagella, have inspired many microswimmers with single or multiple helical flagella [17,18]. These helical flagella overcome the time-reversible Newtonian Stokes flow and generate net positive propulsion [19]. Translocation of protons across the membrane generates the proton motive force, which in turn, provides the energy threshold that allows rotation of the flagellum. When the neighboring flagella rotate in the same direction, they form a bundle that propels the body forward. 

Based on the linearity of the Stokes equations, Cortez et al. introduced the method of regularized Stokeslets [20], which pioneered computational studies to explore helical flagella swimming [21,22,23] and flagellar bundling [24]. Along this line of research, Turner et al. visualized the flagellar motion real-time using fluorescence techniques [25]. Studies that investigate the kinematics and hydrodynamics of flagellar swimmers mostly employ numerical simulations [26,27,28,29,30]. Particularly the resistive force theory (RFT), introduced by Gray and Hancock et al. [31], has been a standard tool to predict the flagellar thrust from normal and tangential drag forces [32,33,34,35,36,37,38,39,40,41,42]. While the RFT calculations are practical for rapid preliminary parametric design of micro-propulsion systems, it cannot provide analysis for the detailed velocity field, the dissipation function and propulsive efficiency. Furthermore, the accuracy of the RFT predictions relies on empirically specified drag coefficients, which can deviate considerably due to the presence of near-by flagella (i.e., bundling), flow regime, wall effects and complex cross-sections [43]. Furthermore, comparison of computational results implementing the RFT method over thrust force measurements for a helical flagellum revealed qualitative and quantitative differences [23]. 

Two-dimensional (2D) particle image velocimetry (PIV) experiments have been primarily employed to study the swimmers with single helical flagellum [44,45]. Despite the progresses towards the understanding of bundle formation and disruption [46,47], the role of the bundled flagella configuration, such as phase difference in improvement of propulsive efficiency, attracted limited attention. Experimental determination of optimal bundling states and the role of phase difference in propulsive efficiency of the bundled flagella have not been studied in the literature to our knowledge, apart from a few computational works [48,49,50]. For instance, Kim et al. employed both a macroscopic scale [51] and scaled-up models [52] incorporating digital imaging and macro PIV respectively to investigate flagellar bundling. These studies highlighted the influence of motor frequency, helix handedness and the sense of motor rotation on the bundling. It was found that the flow field generated by a bundle in a steady state can be approximated as a single rigid helix having twice the filament radius. Although the general flow patterns were successfully quantified, the two-component measurements and single slice data did not allow thrust force or efficiency calculations. As hypothesized in the present manuscript, the phase of bundling can be employed as effective propulsion control element and deserve further research studies.

In the present study, the bacterial PIV experiments are extended to three-dimensions (3D) based on volumetric velocity reconstruction [53]. This approach allowed calculation of thrust force, power loss contribution and hydrodynamic efficiency along the length of a finite flagellum directly from the PIV data. PIV measurements are compared with computational fluid dynamics, direct thrust force measurements and RFT theory for a wide range of Reynolds (Re) numbers. Finally, this approach is applied to analyze multiple flagellar bundling configurations, where the effect of the phase difference between nearby helices on the induced thrust force, energy dissipation and hydrodynamic efficiency are explored simultaneously, the first time in the literature, to our knowledge. 

## 2. Methodology

### 2.1. Experimental Setup and Flagella Configurations

**Figure 1:**
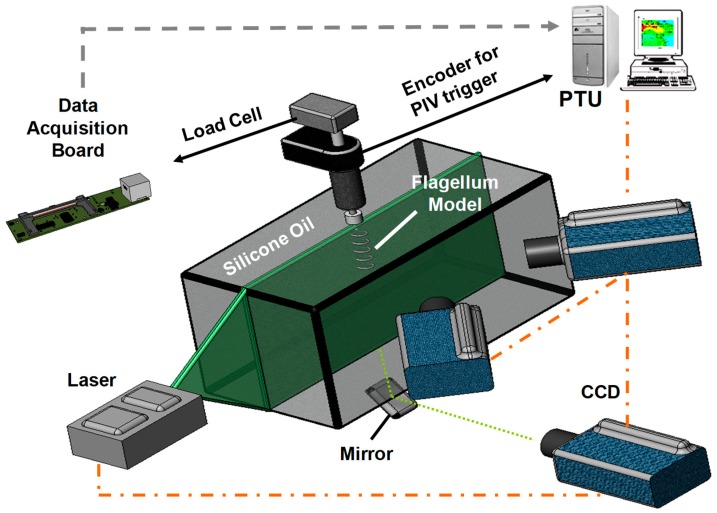
The graphic of the experimental set-up. Only the axial laser sheet configuration is displayed while the lateral 3D particle image velocimetry (PIV) data is acquired for validation and verification purposes using the mirror. PTU: Pulse timing unit.

**Figure 2:**
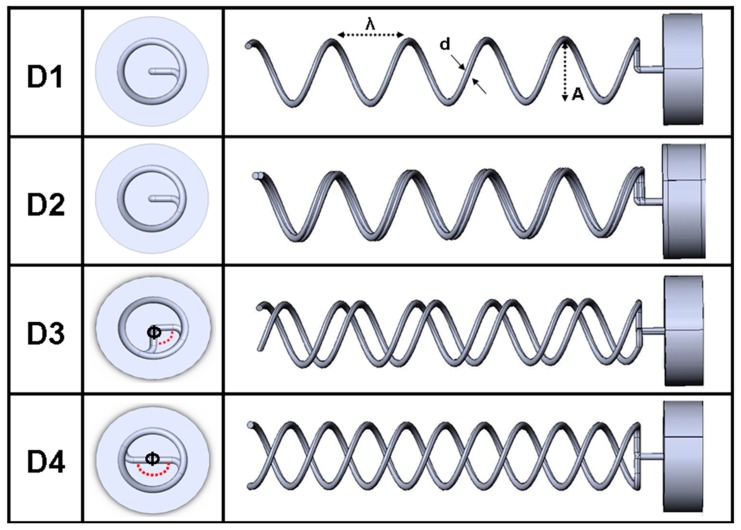
All flagellar configurations tested in the present study. D1: Baseline single-flagellum model. D2: Overlapping bundle state of two nearby flagella. D3: Two flagella with 90-degree relative phase shift. D4: Two flagella with 180-degree relative phase shift. λ =10 mm, A = 3.175 mm.

A vertical static thrust test-bench that allows simultaneous axial thrust load-cell measurements and in-plane lateral or longitudinal 2D/3D PIV velocity acquisition was constructed. A schematic of experimental set-up at various configurations used in this study is presented in Figure 1. Similitude was established between the helical flagella prototype and the full-scale *E. coli* flagella. Helical rigid propulsion elements were fabricated from steel wires wrapped around non-tapered cylindrical rods. The baseline single helix flagellum model had 10 mm pitch length and 6.25 mm helix diameter. Dimensions of all prototype propulsion elements are summarized in Figure 2. Experiments were performed using high (350 cSt) and ultra-high (30,000 cSt) viscosity, µ, silicon oils with a density, *ρ*, of 970 kg/m^3^ (Clearco, Pittsburgh, PA, USA). Re was defined based on the helix diameter and rotational speed and set in the range of 10^–3^ to 10^–2^ according to applied motor speeds. On the basis that a relatively higher Re is realizable for state-of-the art micro-robotic systems, experiments were also extended for Re up to 1. A relatively small size of present set-up (50.8 cm × 25.4 cm × 30.8 cm) eliminated particle mixing problems as previously reported by Kim et al. [52]. A scaled-up helix was placed at the center of a rectangular tank. Pre-experiment computational fluid dynamics (CFD) analysis and earlier experiments ensured that the flow tank was designed with minimal wall influence on the flagella flow field (see Section 2.4).

For simultaneous PIV and direct-thrust force measurements, a 0.1 kg load cell (GSO-100, Transducer Techniques, Temecula, CA, USA) was attached to a cantilever beam holding a mini DC motor (A-max 22 type, Maxon motor, Fall River, MA, USA) coupled to the flagella models through a delrin coupling. The thrust measurement system was characterized in our earlier studies [54,55,56]. Calibration of the load cell was performed using small specific loads where optimal gain and bias of the load cell was adjusted prior to simultaneous PIV measurements. The drive line was first tested by running in air and water to check for possible vibrations and disturbances. Mean deviation of the force was measured to be almost zero (<1%) and the oscillation amplitude, an indicator to assess any source of disturbances, was significantly low (~1 mN) compared to the predicted thrust force values (26–130 mN) using the RFT. During experiments, the thrust force induced by the helix was obtained from the difference between the mean thrust force measured at the motor stop position and the running position in the silicone oil after sufficient time had passed in order to sustain the steady-state conditions of mean values. The actual thrust history was found to be oscillatory in-phase with the rotational speed due to finite-flagella. Six sets of direct force measurements were performed and included for final comparisons with the PIV measurements and RFT approximations. Fabricated prototype single rigid helix was rotated at 1–5 Hz motor speeds range, whereas, 3–7 Hz motor speeds were applied for the experiments with flagellar bundles, i.e., two helices arranged in tandem.

### 2.2. Particle Image Velocimetry (PIV)

A standard PIV system with a Q-switched twin Nd:YAG laser (532 nm, 45 mJ/pulse; Big Sky Laser, Bozeman, MT, USA), a charge-coupled device (CCD) camera (Imager Pro X-2M, Lavision, Ypsilanti, MI, USA) and synchronizer (Lavision, Ypsilanti, MI, USA) was used for recording the particle images. Three-dimensional PIV studies were conducted after performing self-calibration on particle images in addition to the standard calibration procedures with a small calibration target. Images were captured either laterally or axially. For the latter PIV configuration, the laser sheet was perpendicular to the axes of the helices, and a mirror inclined at 45° took images from below. This view was used for the verification of lateral measurements where the laser sheet was aligned with the axes of the helices and images were taken from the front of the tank (see Figure 1). The typical optimal time delay between the two successive pulses was set to be 3000 µs. Laser sheet thickness varied, but was approximately 1.5 mm at the measurement region of interest. To prevent possible light scattering and reflections from the steel wire, 8 µm diameter fluorescent particles (Thermo Fisher Scientific, Waltham, MA, USA), having 300–542 nm excitation and 550–800 nm emission spectrum, were seeded into silicone oil as tracers and compatible filters, with transmission wavelength between 570–1100 nm, were used during PIV recordings.

An optical encoder (USDigital, Vancouver, WA, USA) was introduced to enable trigger signal and gated volumetric PIV recordings (see Section 2.3). All images were recorded with external triggering based on the phase angle signal of the optical encoder. The velocity vectors were calculated applying multi-pass iterations beginning with 32 pixel × 32 pixel interrogation window size down to 16 pixel × 16 pixel interrogation window with adjacent windows overlapping by 50%. Post-processing was performed using the commercial PIV software Davis 7.2 (LaVision. Inc, Gottingen, Germany). The accuracy of the ensemble average of the final vector map was maximized for 70 image pairs for the rotational frequency of interest (see Figure S1 in Supplementary). 

To estimate the uncertainty of the velocity field quantification in our PIV experiment, the relations suggested in the literature [52] was used based on the particle displacement. The RMS (root-mean-square) error of the velocity measurement was calculated based on the relation $\sigma_{u}=\sigma_{\Delta x}/M_{0}.\Delta t$ where Mo is the image magnification and $\sigma_{\Delta x}$ is the RMS error of the displacement on the pixel plane. $\sigma_{\Delta x}$ was defined based on the relation proposed by Raffel. M et al [57] as $\sigma_{\Delta x}=0.04*\left(d_{e}^{2}+d_{r}^{2}\right)$, where $d_{e}$ is the optical diameter of the image prior to being recorded on the pixel plane, and $d_{r}$=7.4 µm represents the resolution of the recording medium, which is considered to be the same as the pixel size. Considering a Gaussian intensity profile together with diffraction-limited particle image, the diameter $d_{e}$ can be mathematically stated as [58]:

(1) $$d_{e}^{2}=M_{0}^{2}\cdot d_{p}^{2}+\left[2.44\cdot \left(1+M_{0}\right)\cdot f^{n}\cdot \lambda \right]$$

Substitution of present experimental data into aforementioned relations (Mo = 0.352, $d_{p}$ = 8 µm, f# = 8 and λ = 532 nm) resulted in the recorded image diameter of 15.87 µm, $\sigma_{\Delta x}$ of 0.311 µm and the RMS velocity measurement error of $\sigma_{u}$ = 0.61 mm/s. This indicates that in our experimental measurements we could resolve velocity values higher than this, which as compared to typical velocity values of 1–2 cm/s is reasonably small. Lateral and axial PIV data acquisition was compared at three axial planes. The differences in radial velocity components were less than 6%. 

### 2.3. Volumetric PIV Reconstruction Protocol for Thrust and Dissipation

**Figure 3:**
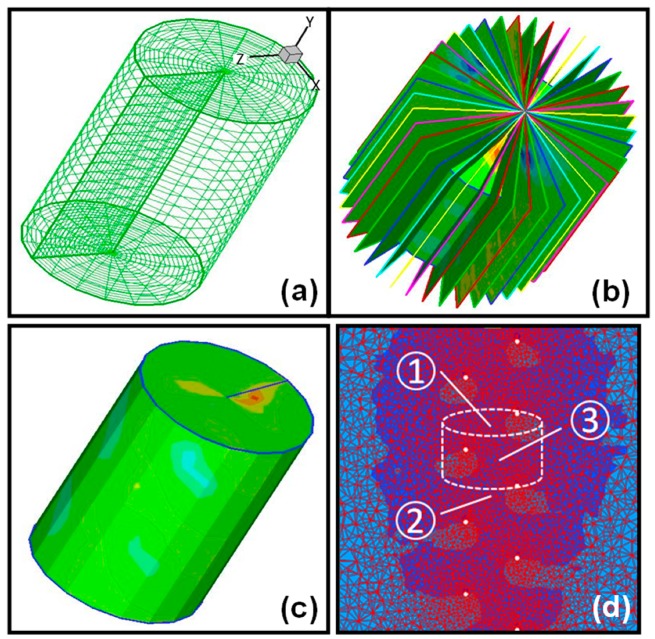
Volumetric PIV data acquisition and analysis methodology. (**a**) Structured C-grid generated for radial (2C/3C) PIV data interpolation. (**b**) 20 PIV slices and (**c**) interpolated volumetric data. (**d**) Sketch of the control volume that spans one pitch of flagella segment for thrust and dissipation calculation and corresponding control surfaces.

Trigger signal from the encoder allowed the acquisition of 2-component (2C) or 3-component (3C) PIV data, at any rotational phase angle without the need to move the cameras or the laser. Our earlier interpolation-based volumetric velocity field reconstruction protocol [53] was modified to incorporate the radially-sliced PIV data acquired in the present study. The augmented slice data was interpolated on a structured cylindrical C-Grid topology (Tecplot, Tecplot Inc., Bellevue, WA, USA) for thrust and efficiency calculations. The methodology used to reconstruct cylindrical volumetric grid is summarized in Figure 3. This approach allowed the reconstruction of the entire 3D velocity field around the rotating flagella. One complete revolution of the helix was divided in to 20 phases (shifting the trigger by 18°). While denser slices would resolve finer 3D flow structures, the optimal number of 20 slices was determined by comparing the results of experiments with denser radial PIV slices. At each phase, the post-processed phase-averaged velocity field of 70 image pairs was exploited (see Figure S1 in Supplementary). 

Reconstructed 3D volumetric data allowed for the definition of a cylindrical control volume of any size and at any axial location containing the flagella, where its segmental thrust force and efficiency could be integrated. A standard control volume spanning two pitch length of the rotating helix model was adopted in this study. The axial component of integral form of the unsteady momentum equation, given in Equation (2), was used to estimate axial thrust values [59]:

(2) $$\int_{\forall }\frac{\partial }{\partial t}\left(\rho \;\vec{V}\right)d\forall +\int_{S}\hat{n}\cdot \rho \vec{V}\vec{V}dS=\int_{S}\hat{n}\cdot \underline{\underline{\sigma }}dS+{\vec{F}}_{flagella}$$

where $\vec{V}$ is the velocity, ρ is the fluid density and ${\vec{F}}_{flagella}$ is the thrust on the flagella. The first term represents the unsteady thrust contribution (due to unsymmetrical flow field generated by the end-effects of finite flagella). Since the entire flow field is time-periodic with the flagella rotational frequency, the axial component of this term is equal to:

(3) $$\frac{\partial V_{y}}{\partial t}d\forall =\frac{\partial V_{y}}{\partial \theta }\cdot \frac{d\theta }{dt}\;d\forall =\frac{\partial V_{y}}{\partial \theta }\cdot \left(rotational\;speed\right)\cdot d\forall$$

Due to periodicity of axial velocity in θ, the volume integral of this term would be zero, which indicates zero contribution of this term in the net thrust force. For Stokes regime, the majority of axial-thrust contribution is originated from the viscous term. However, for future applications with moderate Re the convective and viscous terms can become comparable in magnitude. Axial component of the convective thrust ${\vec{F}}_{flagella}$ that is integrated through the control volume is:

(4) $$\rho \left(V_{x}\frac{\partial V_{y}}{\partial x}+V_{y}\frac{\partial V_{y}}{\partial y}+V_{z}\frac{\partial V_{y}}{\partial z}\right)$$

Whereas the viscous contribution can be calculated from the integral of the following equation:

(5) $$\vartheta \left(\frac{\partial^{2}V_{y}}{\partial x^{2}}+\frac{\partial^{2}V_{y}}{\partial y^{2}}+\frac{\partial^{2}V_{y}}{\partial z^{2}}\right)$$

The flow field around the flagella model, with sufficient distance from the wall, can reasonably be treated as an external flow problem. Likewise, the contribution of pressure difference (dP/dy) term in the stress tensor was estimated as insignificant based on the CFD simulations (maximum 0.1 mN difference along the cylindrical control volume with two pitch length). Most importantly, Equations (2)–(5) indicate that the axial thrust component can be measured exactly from the more practical two-component axially sliced PIV measurements.

The effectiveness of propulsion systems is addressed in several publications [60,61]. The propulsive performance of the helix propeller was estimated from hydrodynamic efficiency definition using the volumetric mean axial velocity of the control volume [62]:

(6) $$\eta =\frac{Useful\;Power}{Useful\;Power+Dissipated\;Power}=\frac{F^{y}{}_{flagella}\cdot V_{axial}}{F^{y}{}_{flagella}\cdot V_{axial}+Dissipated\;Power}$$

The total dissipated hydrodynamic energy along the helix propeller was calculated from the dissipation function, Equation (7), integrated over the control volume [63]:

(7) $$\Phi =\int_{\forall }2\mu \left(\left({\frac{\partial V_{x}}{\partial x}}^{2}+{\frac{\partial V_{y}}{\partial y}}^{2}+{\frac{\partial V_{z}}{\partial z}}^{2}\right)+{\left(\frac{\partial V_{y}}{\partial x}+\frac{\partial V_{x}}{\partial y}\right)}^{2}+{\left(\frac{\partial V_{z}}{\partial y}+\frac{\partial V_{y}}{\partial z}\right)}^{2}+{\left(\frac{\partial V_{x}}{\partial z}+\frac{\partial V_{z}}{\partial x}\right)}^{2}\right)\;d\forall$$

The volumetric radial PIV data reconstruction allowed the calculation of unknown derivatives to evaluate the hydrodynamic efficiency and thrust. The effect of the radial size of the control volume was tested and was found to have negligible effect on the calculated performance parameters reported in this manuscript, as expected. It is worth mentioning that for the RFT drag coefficients we utilized the drag coefficients introduced by Gray and Hancock et al. [31] as:

(8) $$C_{t}=\frac{2\pi \mu }{\ln\left(\frac{2\lambda }{a}\right)-\frac{1}{2}}\;and\;C_{n}=\frac{4\pi \mu }{\ln\left(\frac{2\lambda }{a}\right)+\frac{1}{2}}$$

where $C_{t}$ is the tangential drag coefficient per unit length, $C_{n}$ is the normal drag coefficicent per unit length, λ is the helical flagellum pitch and a is the filament radius.

### 2.4. Computational Fluid Dynamics (CFD) for Experimental Planning

In this study CFD was employed with two major objectives; (i) to guide the design of the test domain, i.e., to verify the dimensions of the rectangular tank where the flagella was placed, and (ii) to validate the experimental approach for estimating the volumetric flow-derived metrics of flagellar propulsion. An iterative design procedure based on the incremental enlargement of the length and width of the tank boundaries identified that the axial flow velocity dissipated 99% in one to four helical diameters away from the center of the rectangular tank (depending on the rotational speed: 1–7 Hz). Hence, the final tank dimensions were determined at least 10 times the axial velocity dissipation length (approximately four helical diameters) in order to ensure minimum wall interference and capture the major flow structures of flagellar propulsion (see Section 3.1).

In order to validate the proposed control volume-based thrust and propulsive efficiency computation, the CFD simulations are performed analogous to the bench-top experiments at multiple rotational speeds and fluid properties. This numerical analysis not only allowed the comparison of PIV and CFD analysis but also identified the effect of tangential velocity component, V_z_, in turn, 2C vs. 3C PIV acquisition on the dissipation function calculations. As discussed in Section 2.3 above, the minimum number of slices required to reconstruct the volumetric flow field and the relative contribution of pressure vs. viscous force to the total flagellar thrust force was also based on the coupled PIV-CFD analysis. This PIV-CFD co-validation study on flagella models was performed for the first time in the literature, to our knowledge. Details of the computational simulations employing the multiple reference frame (MRF) methodology are provided in the Appendix A.

## 3. Results

### 3.1. Single Flagellum Flow Field

The present methodology reconstructs the instantaneous phase-averaged 3D velocity around the rotating flagellar propulsion element. It was observed that the instantaneous flow field appears strongly helical around the flagellum and is characterized by counter-clockwise swirling flow structures, shedding from the flagellum circumferentially. Relatively stagnant flow at the top free surface was diverted downward along the centerline of the flagellum by the vortex tube located beneath the flagellum head and extending downstream along the flagellum length. The velocity field exhibited rapid variations between each pitch. Radial and tangential velocity components (V*_x_* and V*_z_*) displayed a marked increase in close proximity of the flagellum boundaries. This correlates with the direction of helical motion induced by the counter-rotating vortex tube. Axial velocity (V*_y_*) was constant only along the centerline and increased peripherally between each pitch. Momentum in the y-direction was advected by the helical motion, whereas, the suction along the centerline was negligible due to the weak pressure gradient along the length of the flagellum. These characteristic flow features were repeated for each pitch along the flagellum. The variation of velocity magnitude between the head and toe regions was limited to 3%–12% of the velocity magnitude calculated at the middle pitches respectively. 

**Figure 4:**
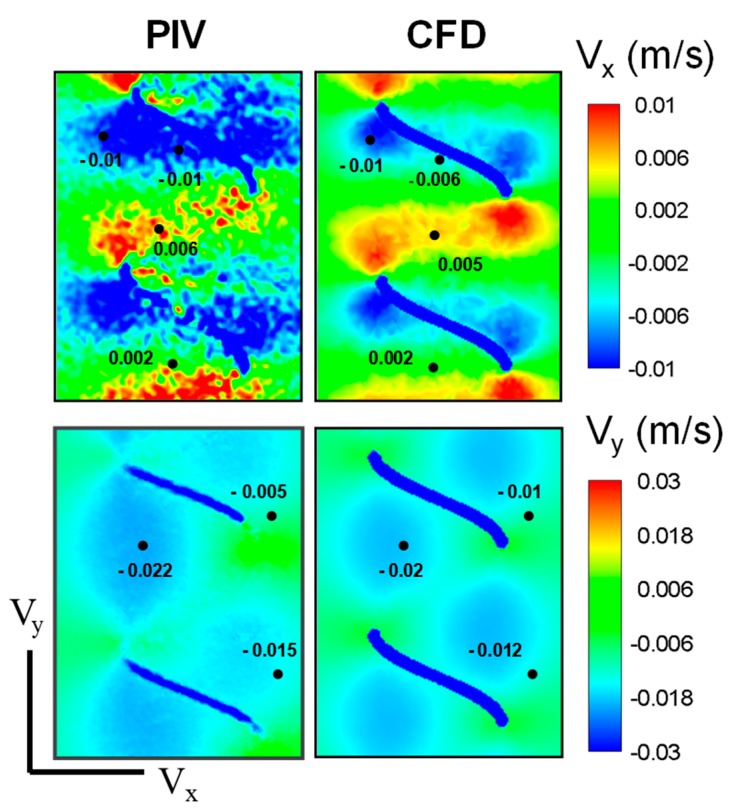
Comparison of radial (V*_x_*) and axial (V*_y_*) velocity components measured and computed with PIV measurements and computational fluid dynamics (CFD) simulations, respectively for D1 (single helix) configuration.

PIV velocity measurement results at 5 Hz were compared with the CFD simulations for both axial and radial velocity components. To perform an identical quantitative comparison with PIV measurements, 1.5 mm laser sheet thickness effect was taken into account in CFD results by averaging 0.75 mm interior and 0.75 mm posterior plane results by inverse distance interpolation. CFD vs. PIV comparisons of the *x*- and *y*-velocity components are shown in Figure 4. Considering the V*_x_* component, CFD simulations and the PIV measurements agreed well, clearly reflecting the induced positive and negative velocity fields along one pitch of the helix. While performing quantitative comparisons, we observed that there was a shadow effect on the negative velocity fields where the helix pierces the laser sheet and partially disrupts the velocity field measurements in that region. Notwithstanding this local perturbation in PIV data, the maximum deviation between PIV-CFD velocity fields is still acceptable in terms of successful agreement, considering both the numerical and experimental complexity of the flagellar propulsion. Likewise, comparing the V_y_ velocity component along the flagellum axis on the frontal plane, there was a strong agreement between the CFD simulation and the PIV results as shown in Figure 4.

Both CFD simulations and PIV measurements highlighted the importance of the induced flow pointing downward in order to provide forward thrust as expected. Quantitative comparison of the velocity field at a number of reference points reveals that CFD predictions and PIV measurements agree within a 20% error margin. In conclusion, both qualitative and quantitative agreement between CFD simulations and PIV measurements co-validates the coupled numerical-experimental methodology employed in this study. 

### 3.2. Velocity Fields Generated by Double-helix Flagella Configurations

**Figure 5:**
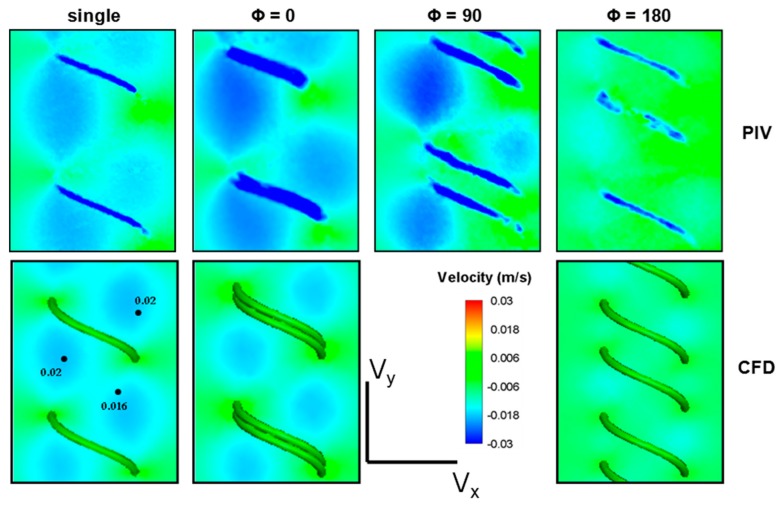
The axial velocity component, V*_y_* in the center plane acquired using PIV measurements (top row) and CFD simulations (bottom row). Compared to the single flagellum, a slight increase is observed for the in-phase (Φ = 0) double flagella configuration. In comparison, the two out-of-phase (Φ = 90 and Φ = 180) double flagella configurations demonstrated a decrease in V*_y_* affecting the thrust production.

In comparison with the single helix flagellum, flow field around three double flagella configurations rotating at 5 Hz were investigated. Both PIV measurements and CFD simulations indicated that the region influenced by the local helical motion was extended considerably after inclusion of the in-phase flagella (see Figure 5). Hence, although the magnitude of the axial discharge velocity remained approximately the same, more flow was directed towards downstream by the in-phase flagella compared to the single flagellum contributing to the axial static thrust force. 

**Figure 6:**
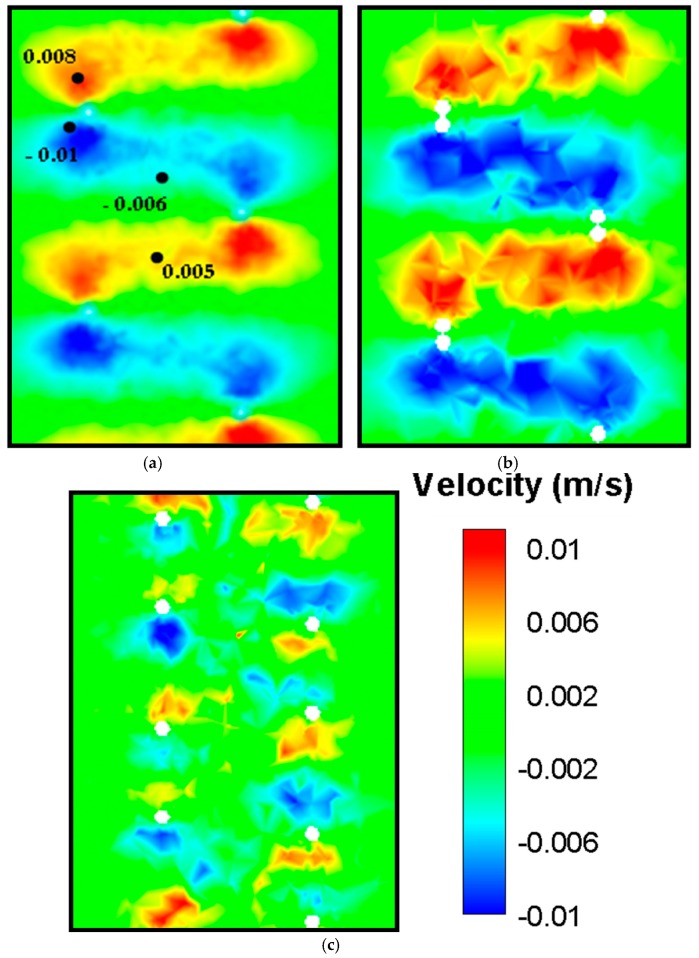
CFD simulations for radial velocity V_x_ plotted in the center-plane, increases from single flagellum (**a**) to in-phase double flagella configuration (**b**) and decreases for the out-of-phase double flagella configuration (**c**).

**Figure 7:**
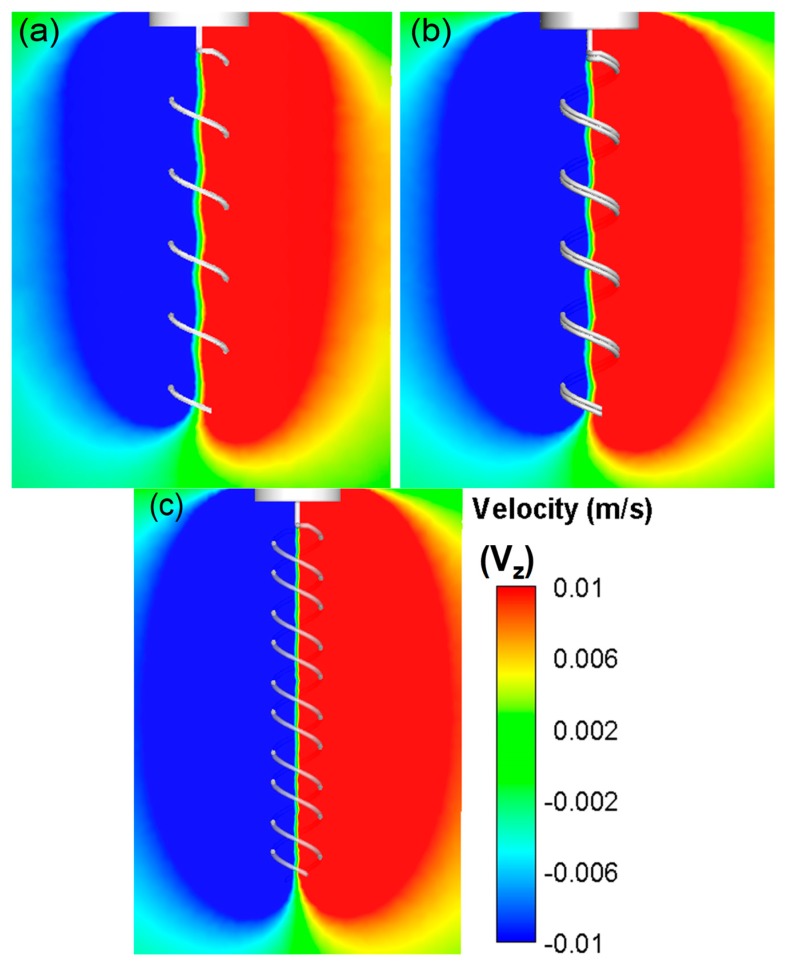
CFD simulations for tangential velocity V_z_ distribution along the center axis of the three major flagella configurations. It shows both inclusion of additional flagellum and phase angle between the flagella increase the flow momentum that is convected in the radial direction. Radial momentum decayed approximately 3, 3^1/2^ and 4 helix diameters away from the single flagellum (**a**), in-phase (**b**) and out-of-phase (**c**) double flagella configurations, respectively.

**Figure 8:**
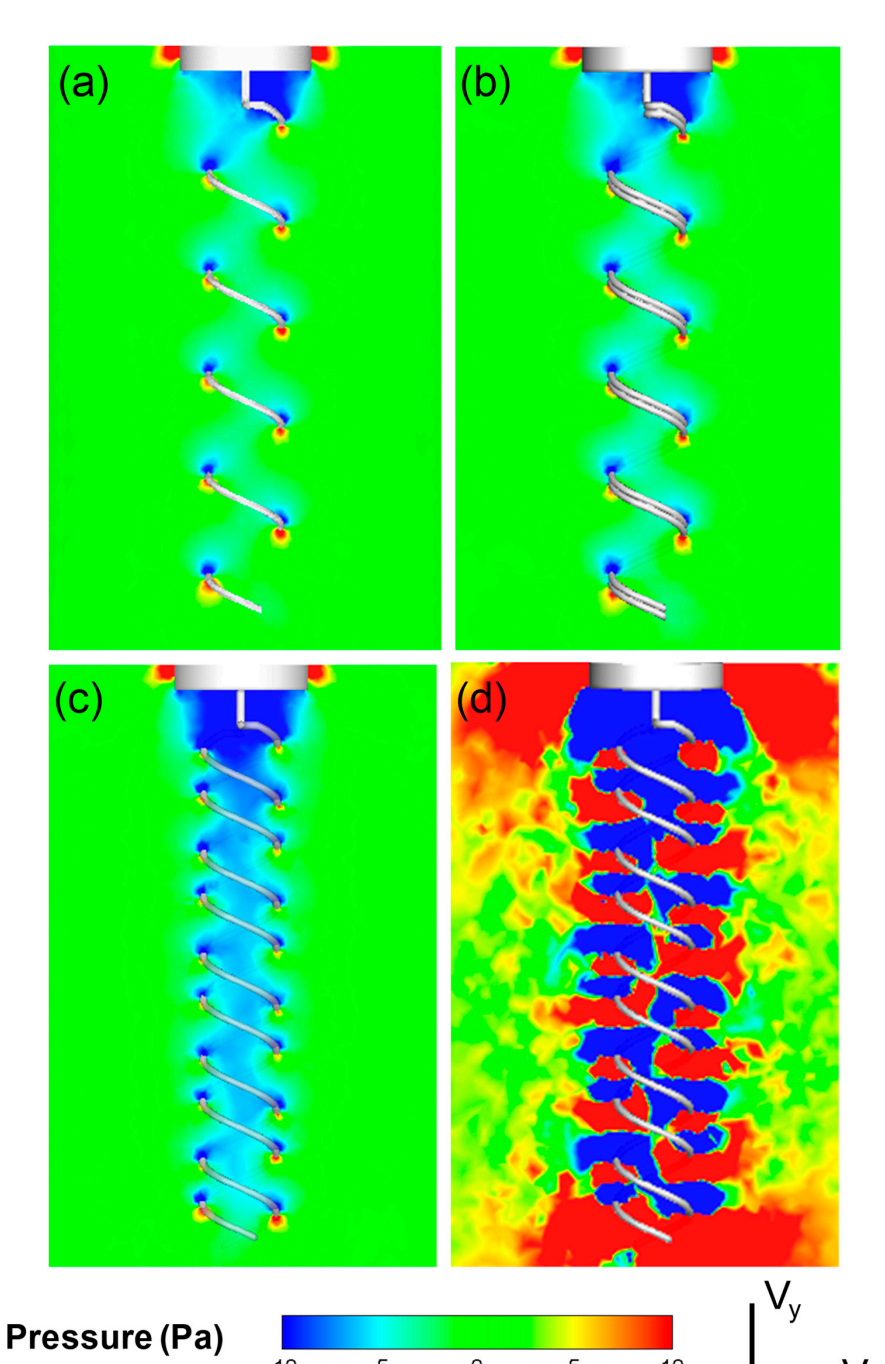
Pressure contours are plotted at the center-plane along the flagella axis for (**a**) single flagellum, (**b**) in-phase, (**c**) out-of-phase double flagella configurations inside less viscous oil (350 cSt) and (**d**) out-of-phase double flagella inside high viscous oil (30,000 cSt). Pressure drop across the flagella increased slightly for the out of-phase configuration and notably for the high viscous case compared to the single flagellum.

For multiple flagella configurations with finite phase, the radial velocity component is considerably complex, especially at the middle section creating periodic cross flow patterns of high and low velocity regions. This complex velocity gradient pattern results in higher dissipation values for the same rotational speed. Furthermore, flow expulsion in the radial direction was increased notably by the double in-phase helix configuration. These patterns were better resolved in CFD simulations (see Figure 6). It was observed that the radial flow velocity increased by 15% to 20%. As the phase angle was introduced, the axial velocity component decreased significantly. On the other hand, the radial velocity of the flow increased significantly as the phase angle between the flagella was increased. Likewise, the tangential velocity component, V_z_, demonstrated a typical vortex pattern that spanned over wider regions as more momentum was convected in the radial direction (see Figure 7). As more flow was directed towards the periphery, the vortex bed was enlarged, which increased the viscous dissipation for phase angled flagella configurations (see Figure 8 — the pressure distribution across the flagella showed a similar contour map as the energy dissipation). These flow patterns agreed well with the thrust and efficiency results that are presented in the next section (see Section 3.3). These results indicated that the phase angle decreases the propulsive efficiency of flagella configurations. These experiments also demonstrated that while variation in the fluid viscosity had little effect on the generated velocity field, it did alter the pressure and viscous forces.

### 3.3. Thrust Force Measured from Volumetric PIV vs. Load Cell

**Figure 9:**
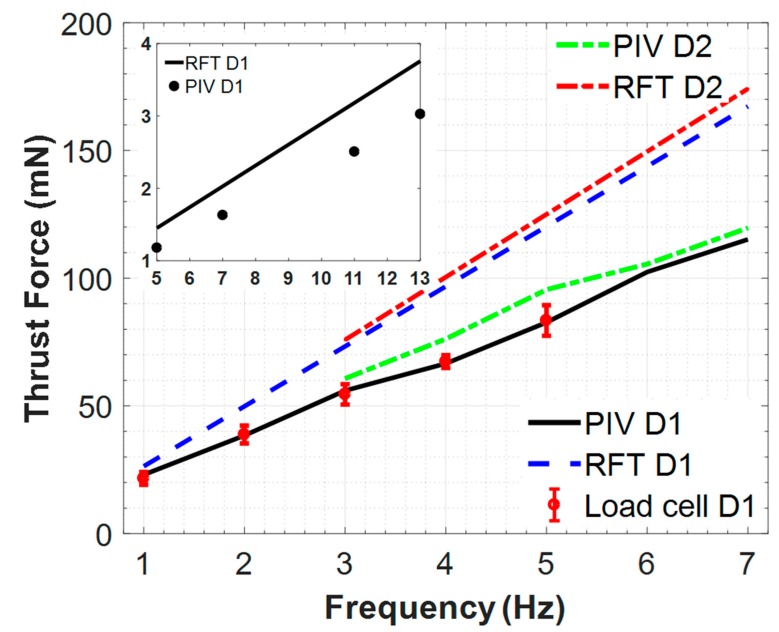
Thrust force comparisons chart: Big chart compares the thrust force for D1 (single) and D2 (overlapping) configurations rotating in 30,000 cSt silicone oil, and the inset chart depicts the thrust force for D1 configuration rotating in 350 cSt silicone oil with the same axes unit as the big chart. Thrust is computed and measured using PIV, load cell measurements and the RFT. Error bars correspond to simultaneous thrust measurements with 20 to 1 confidence level.

In order to validate the control volume-based methodology, simultaneous thrust measurements from load cell data and thrust calculation from the PIV measurements were performed at a dynamic viscosity of 350 cSt and 30,000 cSt. These results are summarized in Figure 9. Thrust calculated through the volumetric PIV protocol was found to be identical to the corresponding simultaneous thrust measurements at all data points for the 30,000 cSt fluid. As expected, thrust values increased linearly with increasing rotational speed. Due to very small loads, the thrust measurements with the fluid of viscosity 350 cSt silicone oil could not be performed with our load cell. However, PIV protocol still provided accurate thrust loads even for these very small Re number regimes. 

### 3.4. Limits of Resistive Force Theory (RFT) Predictions

For all experimental conditions RFT was used to predict the corresponding thrust values (see Figure 9). Even though the general trend is captured accurately with RFT theory, RFT predictions tend to overestimate the actual thrust values by 30% to 55%. As expected, the deviations between RFT and experiments increased linearly with the increasing Re number. For close to biologically-relevant, very small Re numbers that were achieved using 30,000 cSt silicone oil, classical RFT over-predicted the actual thrust as much as 12% (see Figure 9). The accuracy of RFT thrust predictions in bundled flagella configurations is discussed in Section 3.6.

### 3.5. Thrust Variation Along the Axis of a Rigid and Finite Flagellum

The drag coefficients specified in classical RFT assume an infinite flagellum length and ignore the time-dependent complex periodic flow structures at the ends of the flagellum. A realistic finite flagellum model of five pitches was adopted in this study to quantify associated end effects. By shifting the one pitch-length PIV analysis control volume axially, the variations from the average thrust value were computed and found to be approximately 13% at the tip and head compared to the mid-flagellum pitches. The thrust values computed from the mid three pitches varied up to 1%. This variation agrees very well with CFD predictions. 

### 3.6. Double-Helix (Bundling States) Thrust Force Results

The value of the present PIV-based thrust calculation became evident when the accuracy of the RFT thrust predictions were investigated for the serial two flagella configurations. For configurations D3 and D4 with finite phase (see Figure 2), traditional RFT with constant drag coefficients predicted a double increase in thrust compared to the single helix. In contrast, as our measurements indicated, this amplification could not be realized due to the complex flow fields generated (see Section 3.2). The actual thrust of the bi-flagella system was at the same order as the single flagellum. For configuration D2, the overlapping flagella increased the hydraulic diameter, which resulted in a slight increase in the RFT thrust value over the single helix, configuration D1. This slight increase in thrust was also captured using the present PIV methodology (see Figure 9). Note that the standard RFT predictions could not incorporate the effects of complex helix cross-sectional shapes on fluid flow as encountered in configuration D2.

**Figure 10:**
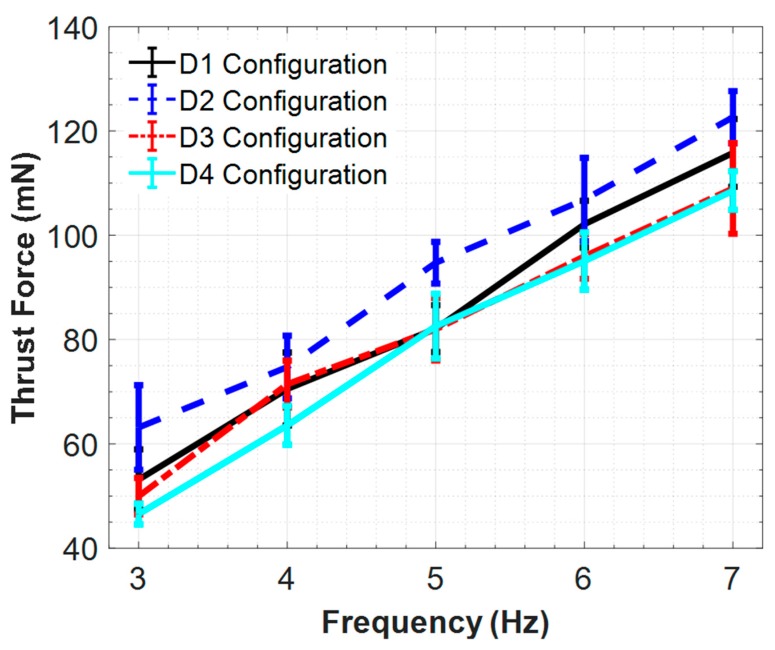
PIV thrust force results of the two bundled flagella for four different configurations in 30,000 cSt silicone oil. Error bars correspond to simultaneous thrust measurements with 20 to 1 confidence level.

Thrust force generated by the bundled flagella for three phase angles are presented in Figure 10. Three sets of PIV experiments were performed for each configuration and the measured values are represented with error bars on this plot in Figure 10. When we examine these results it is concluded that the in-phase helices provide the highest thrust force values while two helices with 180° phase shift in between provide the lowest. Another important inference at this point can be that two helices with 90° and 180° phase shifts in between cannot provide any advantage in thrust force over the single helix. We noticed that single helix and two helices with 90° phase shift in between configurations provided very close thrust force values. Since these two configurations showed similar performance in terms of thrust, we focused on three extreme cases as (i) single, (ii) in-phase and (iii) 180° phase difference configurations and presented the corresponding thrust force results with trend lines. 

We also compared our single helix and in-phase helices results with the RFT predictions, which were valid only for a single flagellum based on the assumption that the in-phase helix as a single propeller had increased thickness compared to the single flagellum. The thickness of the in-phase helix was taken to be 1.4 times more than the single helix thickness by considering the equality of the cross sectional areas (hydraulic diameter). Figure 9 shows these RFT approximations with our PIV method results for single and in-phase helices. For this plot, we used the mean values of three different sets of experimental results, presented in previous figures with error bars. Since there was a slight difference between the PIV method results of the single and in-phase helices, which is similar to what RFT predicts for these two configurations, we concluded that the performance of a flagellar bundle can be approximated as a single propeller where the thickness of the flagellum is increased.

### 3.7. Hydrodynamic Efficiency

**Figure 11:**
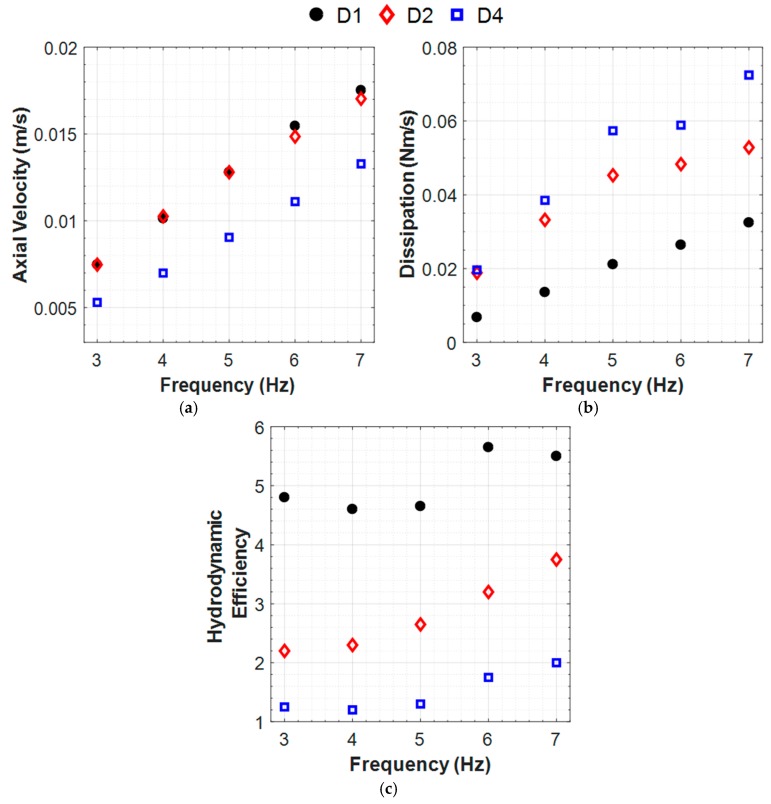
Axial velocity (**a**), energy dissipation (**b**) and hydrodynamic efficiency (**c**) results for different two-flagellum configurations in 30,000 cSt silicone oil.

While the thrust trends can be estimated with reasonable accuracy using RFT calculations, the dissipated energy relies entirely on the velocity field data. For systems that are intended for continuous long-term applications, hydrodynamic efficiency is a key parameter as well as thrust. Hydrodynamic efficiency definition requires consideration of the volume integrated axial velocity component and the total hydrodynamic dissipation function due to the viscous shear (see Equations (6) and (7)). For this study, the hydrodynamic dissipation was computed from both 2D and 3D volumetric PIV reconstruction and found to be different by only 4.5%. The hydrodynamic efficiency was computed for different flagella configurations (see Figure 11). The hydrodynamic efficiency (i.e., the ratio of the useful work to total work for a single flagellum calculated using the PIV measurements) was ~4%–6%, which is comparable with the theoretical estimates at the level of 2%–3%, for rigid helical coil in the literature [37,64]. Figure 11 shows that single helix is the best configuration in terms of hydrodynamic efficiency. Furthermore, in-phase helices were found to provide a lower hydrodynamic efficiency due to the significant increase in dissipated energy, while providing the highest thrust force at the same time. Helices, having certain phase shifts between them (90° and 180°), were also found to fail in efficiency due to the increased energy dissipation.

## 4. Discussion

Flagellar bundling and associated flow structures are complex dynamic processes. Earlier research efforts are mostly limited to sliced planar PIV data and the present study aims to contribute additional information to the literature. Pioneering studies of flagellar bundling have been undertaken using macro-scale models, which suggested that the rate of bundling is proportional to the motor speed and independent of the filament relaxation time [51]. These studies are mostly limited to kinematical observations and the fluid dynamics of bundling states have received limited attention. For example, three-dimensional flow-field around a self-propelled spirochete has been calculated using an immersed boundary Stokeslet method [21]; however, direct experimental flow measurements using gold standard measurement techniques of rotating helical propulsion systems are limited.

In this study, the effectiveness of helical propellers was compared through the hydrodynamic efficiency parameter, which provides a better measure over the thrust force-based performance assessments previously undertaken, especially for longer-term operations. For short-term missions that demand high propulsive power as well, the maximum available thrust for fixed rotational speed becomes critical in relation to the hydrodynamic efficiency. The ratio of useful power to total power generated by the propeller was estimated by considering the dissipated energy losses due to the viscous shearing. While the bundled flagellar systems generated increased thrust force and propulsive power, the inclusion of additional flagella caused significantly higher energy dissipation and lowered the overall hydrodynamic efficiency. The higher the phase angle is between flagella, the lower the energy efficiency is due to reduced propulsive power and increased viscous dissipation. Therefore, our analysis indicates that additional in-phase bundled propeller helices augment the thrust force, yet with a compromised hydrodynamic efficiency.

Both PIV measurements and CFD simulations showed that the flow was induced peripherally and towards downstream of the flagellum during forward thrust generation. Based on the induced velocity fields, the inclusion of the phase angle not only decreased the axial discharge velocity but also caused large local velocity perturbations in the center plane and shunted useless flow towards the radial direction. Both of these flow patterns increased the overall viscous dissipation. It is worth noting that the phase angle decreased the effective pitch length of the flagella up to half of the single flagellum (for 180° case) and decreased the effective propulsion area in between the pitches. Therefore, this paper provides a fluid dynamic explanation to the optimal pitch length (45 degrees), as suggested by other researchers. While the bundled flagella configurations studied here represent a totally different topology compared to the single helix, the effective pitch-length appeared as a major propulsive parameter for the current configurations as well. In other words, it can be deduced that higher sectional area in D1 and complex interplay of flagella in D3 and D4 (where one is in the wake of the other sting) disturbs the flow pattern compared to single flagellum. Furthermore, a higher sectional area not only increases thrust or swept volume, but also increases the viscous dissipation.

In a typical preliminary design scenario of a micro-robot propelled by a flagellar propulsion unit, based on RFT calculations alone to address space/structure constraints, one may incorrectly specify a bundled flagella, instead of a single flagellar propeller that is two times longer. However, our study shows that the propulsive performance of bundled flagellar systems cannot be reliably predicted by traditional RFT that does not consider the complex interactions between the phase-angled flagella units. For the Re range investigated, the inclusion of the additional in-phase flagella increased the thrust force for all rotational speeds. In contrast, a finite phase angle between the flagella caused poor thrust performance. As the phase angle was increased, the thrust force was lowered gradually to the values that are even lower than the single helix baseline configuration. These results clearly indicate that there is a strong interplay between the number of the flagella and their relative helical phase angle, which tightly regulates the resulting thrust force. Our study indicates that the thrust and propulsive efficiency of bundled configurations were significantly reduced compared to single flagellum. This finding applies to natural bacteria propulsion where the bundling configurations are utilized only for temporary durations, similar to a previous study that identified that bundling is utilized for thrust-vector control [65] intermittently. This study also identified bundling efficiency as a major propulsive parameter and recommends future research to investigate the bundling states of bio-propellers in nature. The propulsive efficiency of flagellar configurations measured in this manuscript can be further compared with other low Reynolds number propulsion system candidates, such as jet (or squid) propulsion or undulatory motion [60]. Finding the most efficient method and design for a microswimmer that could be used in future medical applications is still an ongoing research topic. 

Cartesian immersed boundary CFD solvers are routinely employed in contemporary fluid dynamics models of micro-propulsion [49] due to their practical nature; however, this study employs an alternative modeling approach that relies on unstructured grids and rotating coordinate systems. This approach enabled us to control the grid size in the solution domain. Quantitative comparison of velocity field revealed that CFD predictions and PIV measurements are in acceptable agreement within the experimental error margin. Hence, both qualitative and quantitative agreement between CFD simulations and PIV measurements co-validated the coupled numerical-experimental methodology employed in this study. It must be emphasized that CFD results identified more detailed flow structures in the flagella core, which is due to the use of relatively small helix major diameter. One of our major future research objectives is to extend the currently used experimental methodology to flagellar arrays having hundreds of rotating flagellum. Experimental evaluation of such systems requires a manageable scale-up flagellum size. Finally, our PIV experiments incorporated an inherent flexibility, which produced more realistic thrust and efficiency patterns compared to the rigid CFD simulations, as presented in this paper. Both 3D CFD modeling and PIV experiments revealed that the thrust and energy dissipation estimations based on two-component volumetric calculations differed only by 0.3% and 5% from the 3D thrust force and energy dissipation, respectively. This was based on a negligible variation in the tangential (out-of-plane) velocity component, V_z_ along the length of the flagella as proven by the detailed flow analysis. 

Static-thrust testing as employed in this study is a standard tool for evaluating propulsive performance, which has been employed routinely in several macro engineering systems from ship propellers to rocket motors. The effect of free-stream self-propelled velocity is a major limitation of the current study, which can nevertheless be incorporated in to the experimentally validated CFD model fairly easily by introducing an inflow upstream boundary condition. Furthermore, the low Re flow regimes offer analytical approaches that allow for the superposition of different free-stream flow conditions. 

A PIV measurement protocol and volumetric analysis method based on the integration of the unsteady momentum equation was applied in this study to estimate key propulsive performance parameters, such as thrust and hydrodynamic efficiency for flagellar type locomotion, to supplement the visualized flow fields. Both two- and three-component velocity fields were acquired to supplement our understanding of bacterial propulsion hydrodynamics. Acquiring simultaneous thrust force and PIV enabled us to conduct reliable comparisons and test the validity of the proposed approach. By comparing direct force measurement results, RFT predictions and PIV methodology then enabled us to undertake a comprehensive comparison analysis for the single rigid flagellum for a wide range of Re. Strong agreement between the PIV results and the direct force measurements support the utility of the volumetric PIV measurement technique. In these comparisons the end-effects of finite flagella was reduced as much as possible by focusing only on the mid-section of the flagella model both in experiments and RFT computations. However, it is clear that the end effects could still influence the mid-section flow patterns, which are difficult to avoid unless extremely long flagella are used. Present protocol would be particularly useful in complex arrays of propulsion units where the direct measurement of terminal shaft power could be challenging. The discrepancy between the analytic RFT predictions and the PIV/direct force measurements could be further attributed to the geometric imperfections of the hand-made helix propeller and finite material flexibility.

## 5. Conclusions

In order to understand the propulsive mechanics of a single flagellum vs. bundled multiple flagella, a volumetric velocity field reconstruction method based on a minimum of 2D (in-plane) digital PIV velocity components was used in this study. This evaluation procedure was based on the idea of reconstructing a cylindrical control volume, consisting of full field velocity information around the rotating macro-scale flagellum model via PIV experiments. Once the cylindrical control volume was formed, momentum balance equations were applied to quantify the induced thrust while the dissipation function was integrated to calculate the energy losses over any control volume. This measurement protocol was then applied to quantify the propulsive performance parameters of ideal flagellar bundles consisting of two nearby helices rotating in tandem with 0°, 90° and 180° helical phase shifts in between. The performance of a flagellar bundle system with single flagellum performance was analytically compared. This set of experiments confirmed that there is a strong correlation between thrust force, hydrodynamic efficiency and the phase difference between helices. We experimentally and computationally showed that a flagellar bundle, consisting of two in-phase helices, provide slightly better thrust force values that are in agreement with RFT predictions. Hence, the RFT predictions are also very close to each other for single and in-phase helices configurations (as a single propeller). A similar trend was observed using PIV methodology. Finally, these experiments demonstrated that the single helical flagellum is the most efficient configuration for bacteria-inspired microswimmer propulsion. This research study also shows that increased dissipated energy losses for a flagellar bundle system, which was the best option in terms of the thrust force, significantly reduces the overall hydrodynamic efficiency. Through simultaneous force and PIV data acquisition, our experimental process produced reliable comparisons between different flagella configurations. To test the validity and robustness of the proposed technique, multi-modality thrust force analysis was performed incorporating direct force measurement results, RFT predictions and PIV method results for a single rigid flagellum.

## Figures and Tables

**Figure A1:**
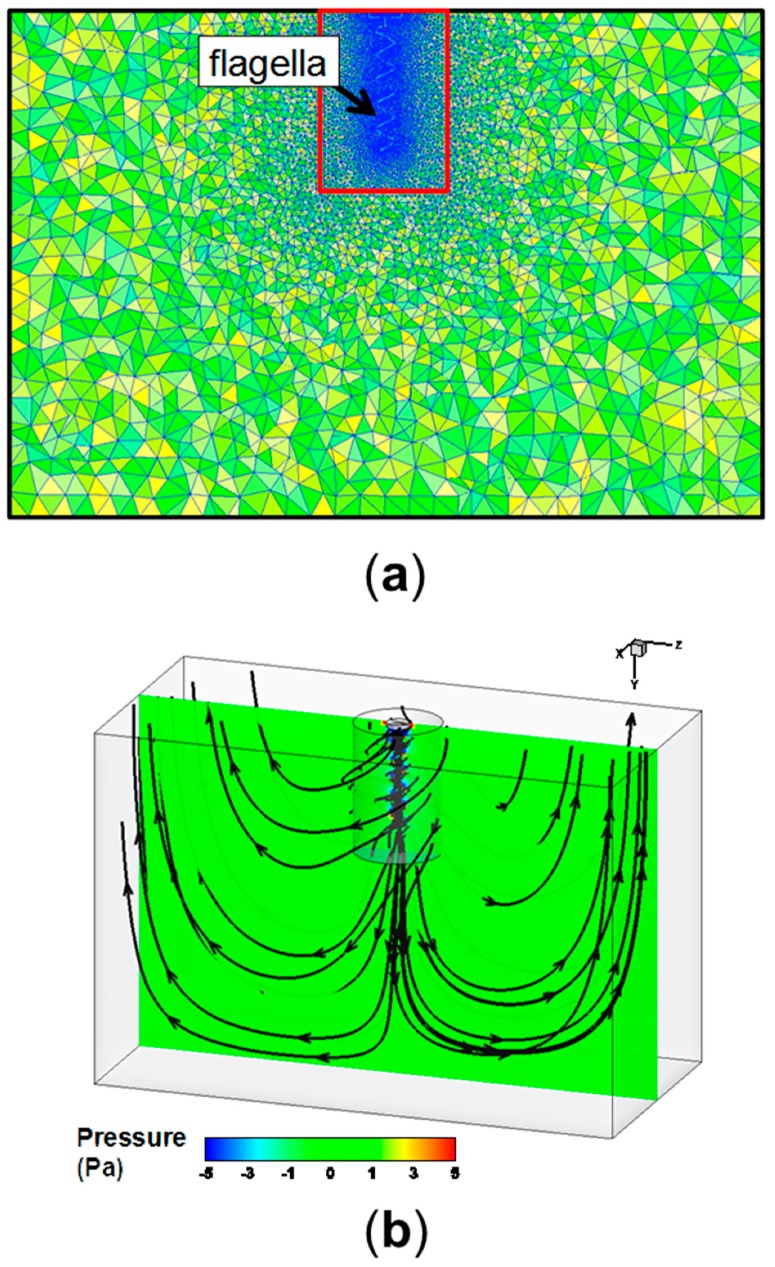
Computational domain for the CFD simulations of the flagella propulsion (**a**) comprised of inner rotating reference frame (red rectangle) and the stationary frame (black rectangle). Helical flow structures around the counter-rotating flagella mapped on the uniform pressure field plotted at the center plane of the flagella (**b**).

## Supplementary Materials

The following are available online at https://www.mdpi.com/2072-666X/10/7/449/s1, Figure S1: Convergence of the ensemble averaging process for velocity field data as a function of the number of image pairs used. Two sets of PIV experiments at different rotational speeds are reported. Each data point represents the total absolute difference between final vector map results by using x and (x-1) image pairs.

## Appendix A. CFD Methodology

In the present study, the 3D CFD model of the bio-mimetic flagella propulsion incorporated the established multiple reference frame (MRF) method, which was employed effectively to simulate flow inside baffled mixing tanks [66,67,68]. The multiple reference frame (MRF) method requires the rectangular tank to be subdivided into two concentric regions; the cylindrical inner subdomain, which contains the flagella and the stationary outer subdomain. The inner subdomain rotates around the center of revolution of the flagella, and is therefore called the moving reference frame. The governing flow equations within the inner subdomain are modified using appropriate velocity transformations resulting in additional acceleration terms. As a result, the overall solution becomes steady with respect to the moving reference frame. At the interface between the rotating and stationary subdomains, the velocity vector and velocity gradients are iteratively exchanged to compute mass and momentum fluxes.Based on the initial CFD simulations, the computational domain is modeled as a rectangular tank with dimensions indicated in Section 2.1. The inner subdomain radius is approximately 4 helical diameters (2.5 cm) to minimize the flow interactions at the domain interfaces, which ensures the robustness of the MRF method. In order to ensure the appropriate grid resolution around the flagella and also to decrease the element count at the relatively unimportant regions (close to the tank walls), gradual refinement and coarsening methods are employed within the inner and outer subdomain respectively. As a result, the inner and outer subdomains are discretized with 740 k and 480 k tetrahedral elements, respectively (see Figure A1) using the grid generation software GAMBIT (ANSYS Inc., Canonsburg, PA, USA). Moreover, strict mesh quality checks are applied throughout the domain in order to improve the performance of the model to predict the helical flow structures around the flagella.

The CFD model incorporates a steady second order accurate segregated solver in FLUENT version 6.3.26 (ANSYS Inc., Canonsburg, PA, USA) to simulate incompressible Stokes flow for two oil viscosities: 350 cSt and 30,000 cSt. The built-in semi-implicit method for pressure linked equations (SIMPLE) is employed in order to ensure mass conservation and obtain the pressure field. PRESTO (pressure staggering option) scheme, which is well-suited for the steep pressure gradients involved in rotating flows, is set as the pressure interpolation method. Derivatives are evaluated by the node-based gradient method to ensure second order spatial accuracy for the unstructured computational grid. The top surface of the domain is assigned as a pressure inlet with zero gage pressure and the remaining surfaces are treated as rigid walls with no-slip boundary condition. The convergence is achieved in multiple steps; first by obtaining a preliminary solution with an Euler solver at low rotational speeds (i.e., full multigrid initialization), preceding an intermediate solution using the first order discretization methods. The final solution is achieved by setting the latter as an initial condition to advance the second order accuracy and gradually increasing the rotational speed of the inner subdomain to the desired flagellum speed. The convergence is monitored by means of the flow monitors assigned at both the inner and outer domains. In addition, the residual of continuity equation is reduced at least to 10^−8^. Computations took approximately 8 h to reach final solution using a Linux work station with two Quad Core Intel Xeon Processors (8 nodes each 2.66 GHz) with 8 GB of shared parallel memory.

It is worthwhile to note that based on the steady-state form of the governing transport equations, MRF required less computational time compared to the alternative moving wall approaches [67]. Still, the MRF method provided high accuracy as the nominally Stokes flow regime eliminated strong circumferential interactions at the sub domain interface and the subdomain interface was modeled sufficiently away from the helical flow.
