# Supplementary material for: Thrust and Hydrodynamic Efficiency of the Bundled Flagella
Source: Micromachines (Basel). 2019 Jul 4;10(7):449. doi: 10.3390/mi10070449 (PMC6680724; doi:10.3390/mi10070449)
Supplement: Supplementary file 1 [file micromachines-10-00449-s001.pdf]

Supplementary Materials: Thrust and Hydrodynamic Efficiency of the Bundled Flagella

Umit Danis [1], Reza Rasooli [2], Chia-Yuan Chen [3], Onur Dur [4], Metin Sitti [1,2,5], and Kerem Pekkan [2,4,*]

[1] Department of Mechanical Engineering, Carnegie Mellon University, Pittsburgh, PA 15213, USA
[2] Department of Mechanical Engineering, Koc University, Istanbul 34450, Turkey
[3] Department of Mechanical Engineering, National Cheng Kung University, Tainan 701, Taiwan
[4] Department of Biomedical Engineering, Carnegie Mellon University, Pittsburgh, PA 15213, USA
[5] Physical Intelligence Department, Max Planck Institute for Intelligent Systems, Stuttgart 70569, Germany
* Correspondence: kpekkan@ku.edu.tr; Tel.: +90-(533)-356-3595; Fax: +90-(212)-338-1548

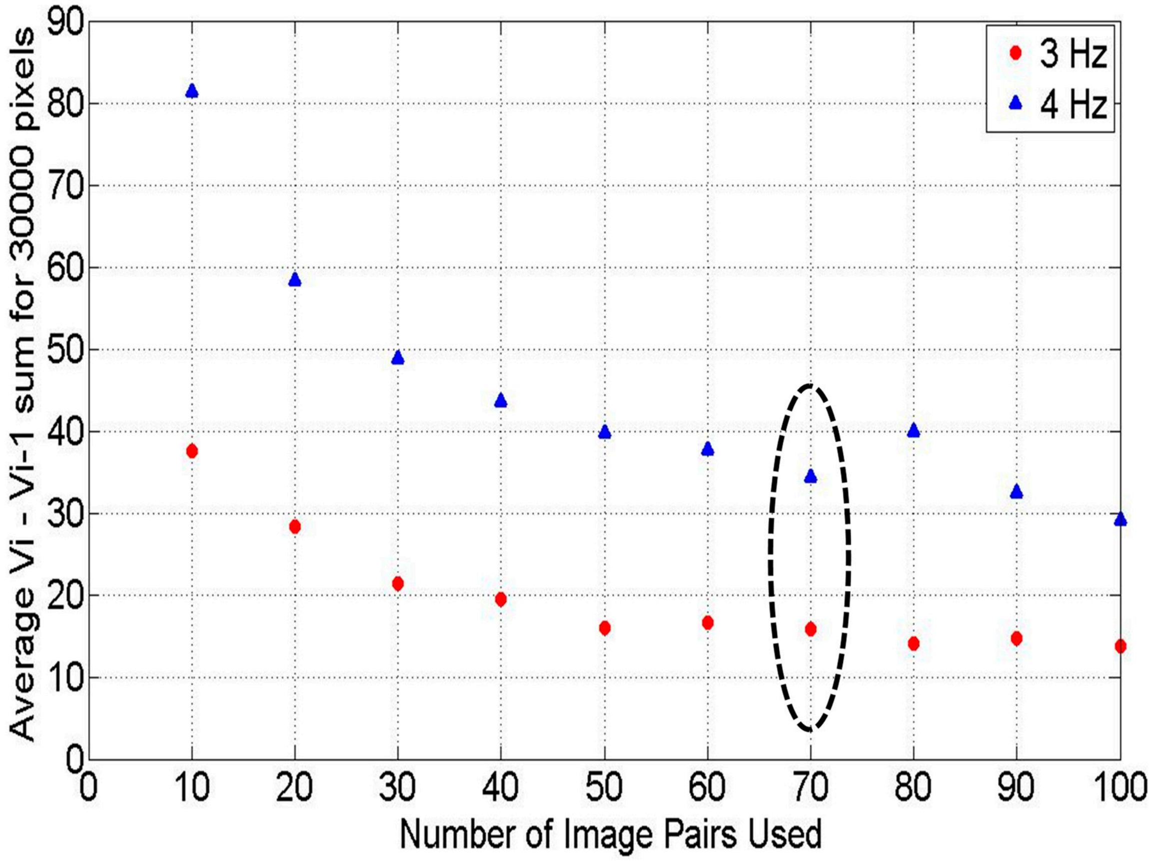

Figure S1. Convergence of the ensemble averaging process for velocity field data as a function of the number of image pairs used. Two sets of PIV experiments at different rotational speeds are reported. Each data point represents the total absolute difference between final vector map results by using x and $(x-1)$ image pairs.
